# Gaseous Air Pollutant Exposure and Atherosclerosis: A Systematic Study Integrating Multi-Omics and Network Toxicology

**DOI:** 10.3390/ijms27167381

**Published:** 2026-08-18

**Authors:** Husileng Hai, Qianhe Wang, Juan Li, Jie Wang, Xijuan Jiang, Maojuan Guo

**Affiliations:** 1 School of Traditional Chinese Medicine, Tianjin University of Traditional Chinese Medicine, Tianjin 301617, China; 13190952835@163.com; 2 School of Integrative Medicine, Tianjin University of Traditional Chinese Medicine, Tianjin 301617, China; qianhesss@163.com (Q.W.); 17302275789@163.com (J.L.); wangjie416@tjutcm.edu.cn (J.W.)

**Keywords:** atherosclerosis, gaseous air pollution, single-cell sequencing, metabolic reprogramming, inflammation

## Abstract

Gaseous air pollution is a major environmental risk factor for atherosclerosis, yet the specific molecular networks linking Gaseous air pollution to atherosclerosis are not fully characterized. This study aimed to elucidate how gaseous pollutants drive atherosclerosis using a multi-level framework integrating network toxicology, nine machine-learning algorithms, bulk and single-cell/spatial transcriptomics, molecular docking, clinical tissue/serum validation, and in vitro pollutant-exposure assays. A conserved *RXRA*–*MMP9*–*TNF* axis was identified as a core molecular network linking pollutant exposure to atherosclerosis. Single-cell and spatial transcriptomics showed broad expression of *RXRA* (*Retinoid X Receptor Alpha*) in vascular stromal and endothelial cells and its association with xenobiotic and lipid metabolism, whereas *MMP9* (*Matrix Metallopeptidase 9*) and *TNF* (*Tumor Necrosis Factor*) were enriched in macrophages, T cells, and mast cells within plaques, correlating with immune cell infiltration. Multiple pollutants displayed high binding affinity to these proteins. Clinical samples showed upregulation of these targets in atherosclerotic tissue and serum. Toluene exposure in THP-1-derived macrophages significantly increased *RXRA*, *MMP9*, and *TNF* mRNA and protein levels. These findings highlight an immune–metabolic interplay centered on the *RXRA*–*MMP9*–*TNF* axis in Gaseous air pollution-driven atherosclerosis, supporting these molecules as candidate biomarkers for environmental health strategies.

## 1. Introduction

Atherosclerosis (AS) and its related cardiovascular and cerebrovascular diseases represent a leading cause of global mortality [1]. The pathogenesis of AS involves complex interrelated processes. In the early stages, endothelial injury [2], dyslipidemia [3], and hemodynamic disturbances initiate the disease [4]. As AS progresses, macrophage infiltration and excessive secretion of matrix metalloproteinases (MMPs) accelerate plaque destabilization, which can ultimately lead to rupture and acute cardiovascular events [5,6].

In addition to traditional risk factors, gaseous pollutants, including benzene, sulfur dioxide (SO_2_), nitrogen dioxide (NO_2_), ozone (O_3_), nitric oxide (NO), carbon monoxide (CO), and toluene, are now recognized as significant environmental contributors to AS [7]. Long-term exposure to these pollutants is associated with an increased risk of AS progression and cardiovascular events [8]. Mechanistically, pollutants exacerbate AS by inducing endothelial dysfunction, chronic inflammation, oxidative stress, and metabolic dysregulation [9,10,11].

The seven gaseous pollutants investigated in this study are prevalent in urban and/or industrial environments. Benzene and toluene are volatile organic compounds (VOCs) primarily emitted from vehicle exhaust, fuel evaporation, and industrial solvents. SO_2_ and NO_2_ originate largely from fossil fuel combustion in power plants and vehicles, and in the atmosphere they undergo further oxidation to form sulfate and nitrate particles, contributing to secondary PM_2.5_ formation. Ground-level O_3_ is a secondary pollutant generated through photochemical reactions of NO_x_ and VOCs in the presence of sunlight. CO and NO are products of incomplete combustion and serve as indicators of traffic-related gaseous air pollution. It should be noted that some of these compounds, such as toluene and benzene, are found at relatively low concentrations in ambient air under typical environmental conditions; their health effects have been primarily documented in occupational settings or in scenarios with specific pollution sources. Beyond their well-documented respiratory effects, long-term exposure to these pollutants has been linked to systemic health outcomes, including increased risks of cardiovascular mortality, stroke, and metabolic disorders. Understanding the molecular pathways through which these pollutants may exacerbate cardiovascular disease is therefore of substantial public health importance.

Despite extensive research, how these established pathological processes are molecularly connected to specific pollutant exposures across different cell types and spatial contexts remains incompletely defined. Recent advancements suggest that gaseous pollutants influence vascular disease, in part, through epigenetic modifications of metabolic and immune genes, thereby impairing vascular function [12]. Specifically, pollutants may induce metabolic reprogramming in macrophages, enhancing their pro-inflammatory polarization and amplifying inflammation [13]. These findings extend the oxidative stress–inflammation model by incorporating the metabolic-immune interplay that may be targeted by pollutants. However, questions remain regarding how pollutants shape the vascular microenvironment at both the cellular and spatial levels, which molecular hubs mediate these changes, and how various cell types contribute to pathological outcomes.

Based on the above, the present study aimed to investigate the molecular links between air pollutant exposure and atherosclerosis using a multi-level framework. Specifically, we employed network toxicology and machine learning to identify key pathogenic genes within pollutant–gene interaction networks. Single-cell and spatial transcriptomics were then utilized to localize their cellular origins and tissue distribution. Finally, clinical histopathology, serological analysis, molecular docking, and in vitro pollutant exposure were integrated to validate the expression characteristics and relevance of these molecules. This study sought to provide a systematic molecular perspective on how Gaseous air pollution may exacerbate atherosclerosis. This study sought to provide a systematic molecular perspective on how gaseous pollutants may exacerbate atherosclerosis, focusing on seven gaseous pollutants—benzene, SO_2_, NO_2_, O_3_, NO, CO, and toluene—selected for their defined chemical structures, atmospheric relevance to PM_2.5_, and experimental feasibility.

## 2. Results

### 2.1. Analysis of Targets Associated with Gaseous Air Pollution-Induced Atherosclerosis

To identify potential molecular links between pollutant exposure and atherosclerosis (AS), we applied an integrated computational approach using four prediction platforms (TargetNet, ChEMBL, STITCH, and SuperPred). This analysis yielded 744 putative targets associated with seven gaseous pollutants. The target distribution was as follows: Benzene (74), SO_2_ (246), NO_2_ (528), O_3_ (88), NO (92), CO (184), and Toluene (87) (Supplementary Table S1). Following standardization and redundancy removal, this target set was used for subsequent analysis.

**Figure 1:**
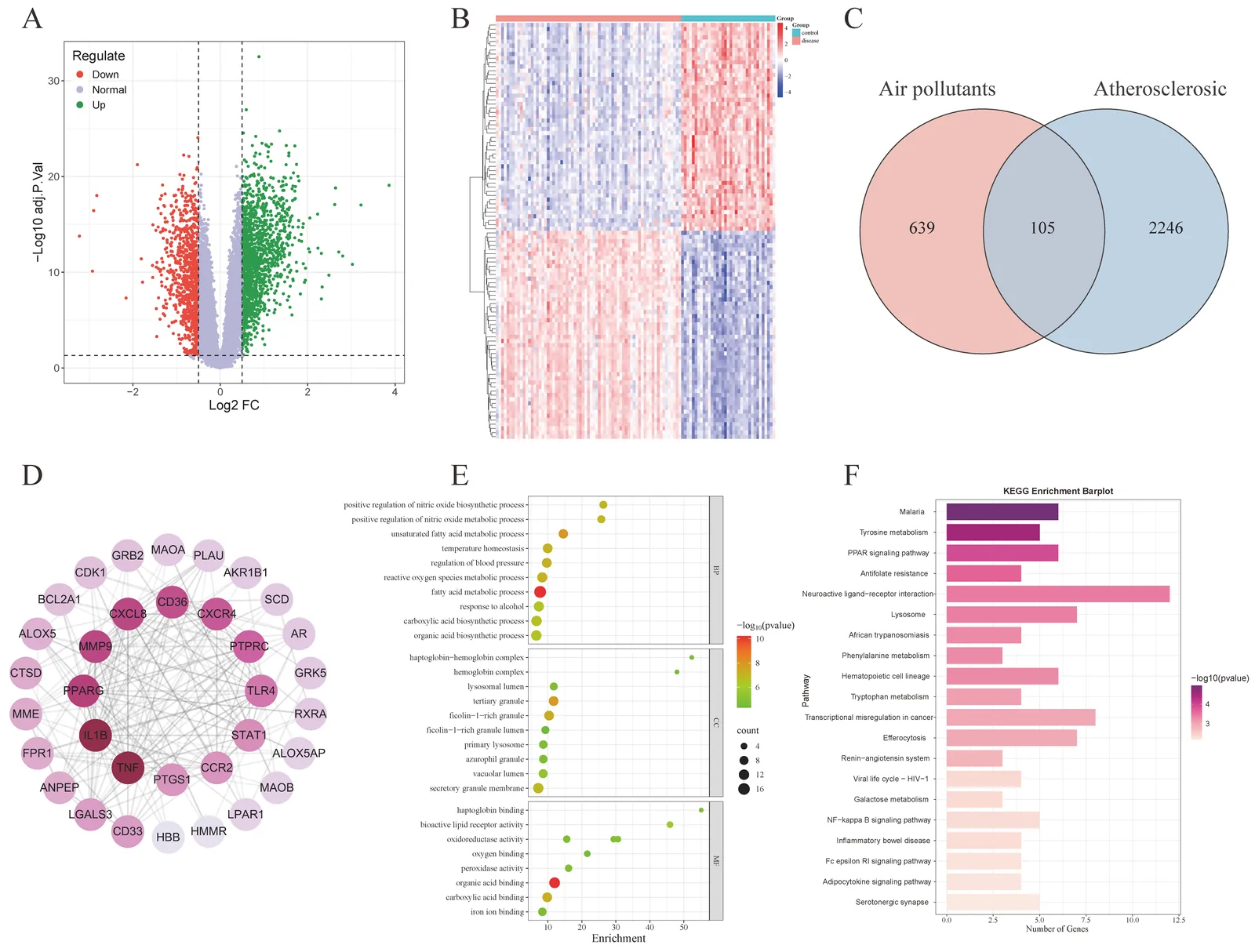
Analysis of Targets Associated with Gaseous Air Pollution-Induced Atherosclerosis. (**A**) Red and blue colors represent significantly up-regulated and down-regulated differentially expressed genes (DEGs) in the AS group, respectively; (**B**) Heatmap displaying the top 50 significantly up- and down-regulated genes in the AS and control groups; (**C**) Overlapping targets between gaseous pollutants and atherosclerosis; (**D**) PPI network of the 34 hub genes. Nodes represent DEGs, and edges represent interactions between nodes; (**E**) GO analysis; (**F**) KEGG analysis.

Based on transcriptomic profiling of the GSE100927 dataset (69 atherosclerosis patients and 35 healthy controls), our systematic analysis identified 2351 differentially expressed genes (DEGs) associated with atherosclerosis using thresholds of |log_2_FC| ≥ 0.5 and adjusted *p*-value < 0.05. Among these, 1428 genes were up-regulated while 923 were down-regulated (Figure 1A,B). This robust set of DEGs establishes a comprehensive molecular foundation for further investigation into atherosclerosis pathogenesis.

The intersection between air pollutant-related targets and atherosclerosis-associated differentially expressed genes revealed 105 overlapping genes (Figure 1C). We identified these as key candidate targets that may mediate the pro-atherosclerotic effects of gaseous pollutants. Next, we constructed a protein–protein interaction (PPI) network using the STRING database and analyzed its topological features with the CytoNCA plugin in Cytoscape 3.9.1. Applying a stringent selection criterion, we required above-median values for six centrality measures—betweenness (BC), closeness (CC), degree (DC), eigenvector (EC), network (NC), and local average connectivity (LAC). This analysis identified 34 hub targets (Figure 1D) that play central roles in the network architecture.

We systematically characterized the biological functions and potential mechanisms of the 105 overlapping genes through comprehensive Gene Ontology (GO) annotation and KEGG pathway enrichment analyses. GO analysis revealed significant enrichment in biological processes, including nitric oxide metabolism, fatty acid metabolic processes, and peroxidase activity (Figure 1E). Concurrently, KEGG pathway analysis demonstrated prominent involvement in the NF-κB signaling pathway and adipocytokine signaling pathway (Figure 1F), both of which are critically involved in inflammatory responses and metabolic regulation. GO and KEGG enrichment analyses of the 105 overlapping genes revealed significant involvement of inflammatory response and lipid metabolism-related pathways.

### 2.2. Machine Learning Screening Identifies Core Genes

**Figure 2:**
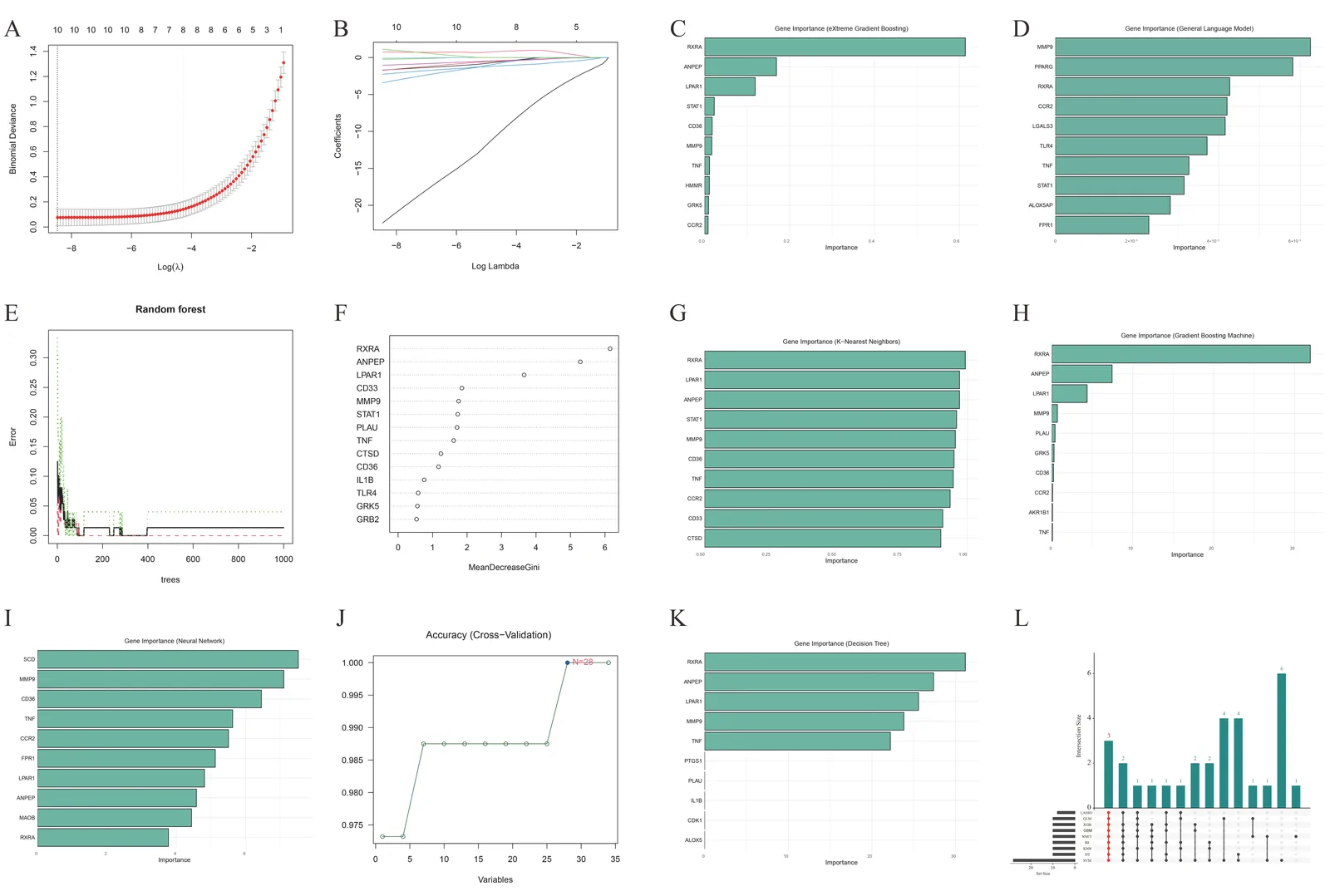
Machine Learning-Based Identification of Core Genes. (**A**,**B**) LASSO algorithm; (**C**) XGBoost algorithm; (**D**) NNET algorithm; (**E**,**F**) Random Forest algorithm; (**G**) KNN algorithm; (**H**) GBM algorithm; (**I**) GLM algorithm; (**J**) SVM algorithm; (**K**) DT algorithm; (**L**) UpSet plot showing the feature genes shared across the nine algorithms.

We systematically identified core targets in gaseous air pollution-induced atherosclerosis by integrating nine distinct machine learning algorithms for robust feature gene selection. Specifically, LASSO regression selected 10 feature genes, while the support vector machine (SVM) algorithm identified 28 feature genes. The remaining seven algorithms—including Random Forest and XGBoost—each provided their top 10 importance-ranked genes (Figure 2A–K). To prioritize genes consistently identified across algorithms, we applied UpSetR visualization to analyze intersections among the nine gene sets (Figure 2L). This consensus analysis identified *RXRA*, *MMP9*, and *TNF* as candidate core genes for further investigation.

### 2.3. Immune Infiltration Analysis

**Figure 3:**
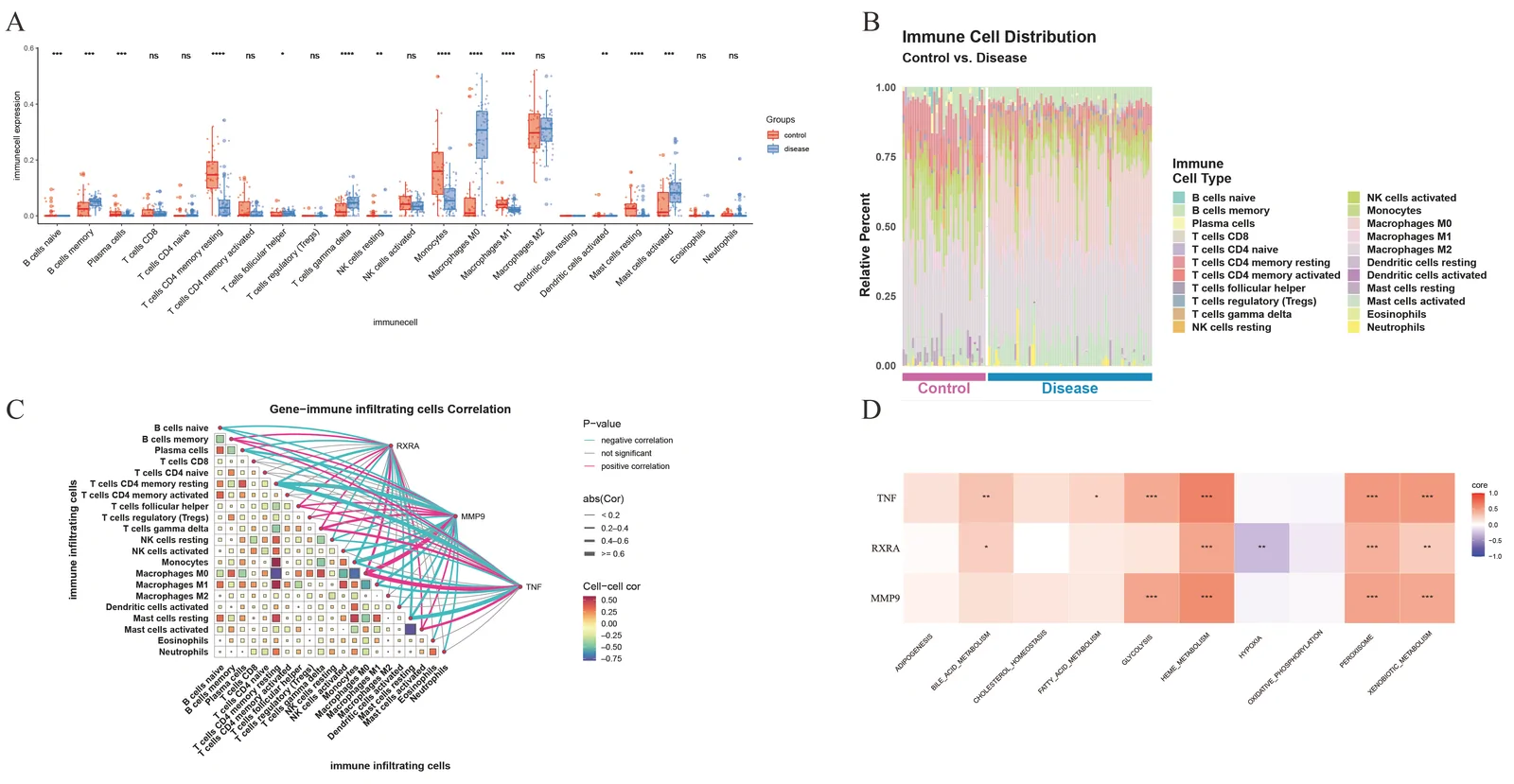
Immune infiltration and metabolic pathway correlation analysis in atherosclerosis. (**A**) Box plot showing the relative abundance of 22 immune cell types between atherosclerosis (AS) and control samples. * *p* < 0.05, ** *p* < 0.01, *** *p* < 0.001, **** *p* < 0.0001; ns, not significant; (**B**) Bar chart displaying the infiltration levels of 22 immune cell types across individual samples; (**C**) Correlation heatmap between core genes (*RXRA*, *MMP9*, *TNF*) and immune cell types. Pink lines indicate significant positive correlations, blue lines indicate significant negative correlations; line thickness represents correlation strength; (**D**) Heatmap of Gene Set Variation Analysis (GSVA) showing correlations between core gene expression and metabolic pathways. Red and blue represent positive and negative correlations, respectively. * *p* < 0.05, ** *p* < 0.01, *** *p* < 0.001.

Recognizing the established link between immune regulation and atherosclerosis (AS) pathogenesis, we applied the CIBERSORT algorithm to characterize the infiltration patterns of 22 immune cell types in AS specimens. Our analysis revealed significantly elevated infiltration of activated mast cells in atherosclerotic lesions compared to normal controls (Figure 3A). Conversely, we detected substantial reductions in naïve B cells, plasma cells, resting CD4+ memory T cells, and resting mast cells. The comprehensive infiltration profiles of all 22 immune cell subtypes are presented in Figure 3B, illustrating the profoundly altered immune landscape in atherosclerosis.

Specifically, the AS group exhibited markedly increased infiltration of macrophages, activated mast cells, and neutrophils, alongside significantly decreased proportions of resting CD4+ memory T cells and resting mast cells.

We conducted Spearman correlation analysis to examine associations between the three core genes and peripheral immune cell infiltration and to identify common regulatory patterns. As shown in Figure 3C, all three genes exhibited significant positive correlations with M0 macrophages and activated mast cells, along with significant negative correlations with resting CD4+ memory T cells and resting mast cells. This consistent correlation pattern indicates that the core genes collectively modulate the activation states of myeloid-derived cells and mast cells, thereby contributing to immune microenvironment dysregulation in atherosclerosis. Our findings reveal a coordinated association between these genes and the altered immune cell landscape in atherosclerosis, suggesting their collective involvement in the dysregulated immune microenvironment.

### 2.4. GSVA Reveals Metabolic Pathways Correlated with Core Genes

We systematically evaluated the role of the core genes in metabolic reprogramming using Gene Set Variation Analysis (GSVA) across 50 hallmark pathways, focusing on 10 metabolism-related pathways (Figure 3D). Subsequently, Spearman correlation analysis revealed significant positive associations (*p* < 0.05) between the three core genes (*MMP9*, *TNF*, and *RXRA*) and three key metabolic pathways: heme metabolism, xenobiotic metabolism, and the peroxisome pathway. These consistent correlations suggest that the core genes critically regulate oxidative stress response and detoxification processes. Our findings indicate that gaseous pollutants may promote atherosclerotic pathogenesis by disrupting these essential metabolic processes. Specifically, enhanced redox cycling and xenobiotic metabolism may contribute to intracellular oxidative stress and inflammatory activation. This observation provides additional molecular context for the recognized connection between pollutant exposure, metabolic dysregulation, and vascular inflammation, offering a framework for understanding how environmental triggers may engage metabolic-immune crosstalk in atherosclerosis.

### 2.5. Molecular Docking Reveals Binding Affinities of Pollutants to Core Targets

**Figure 4:**
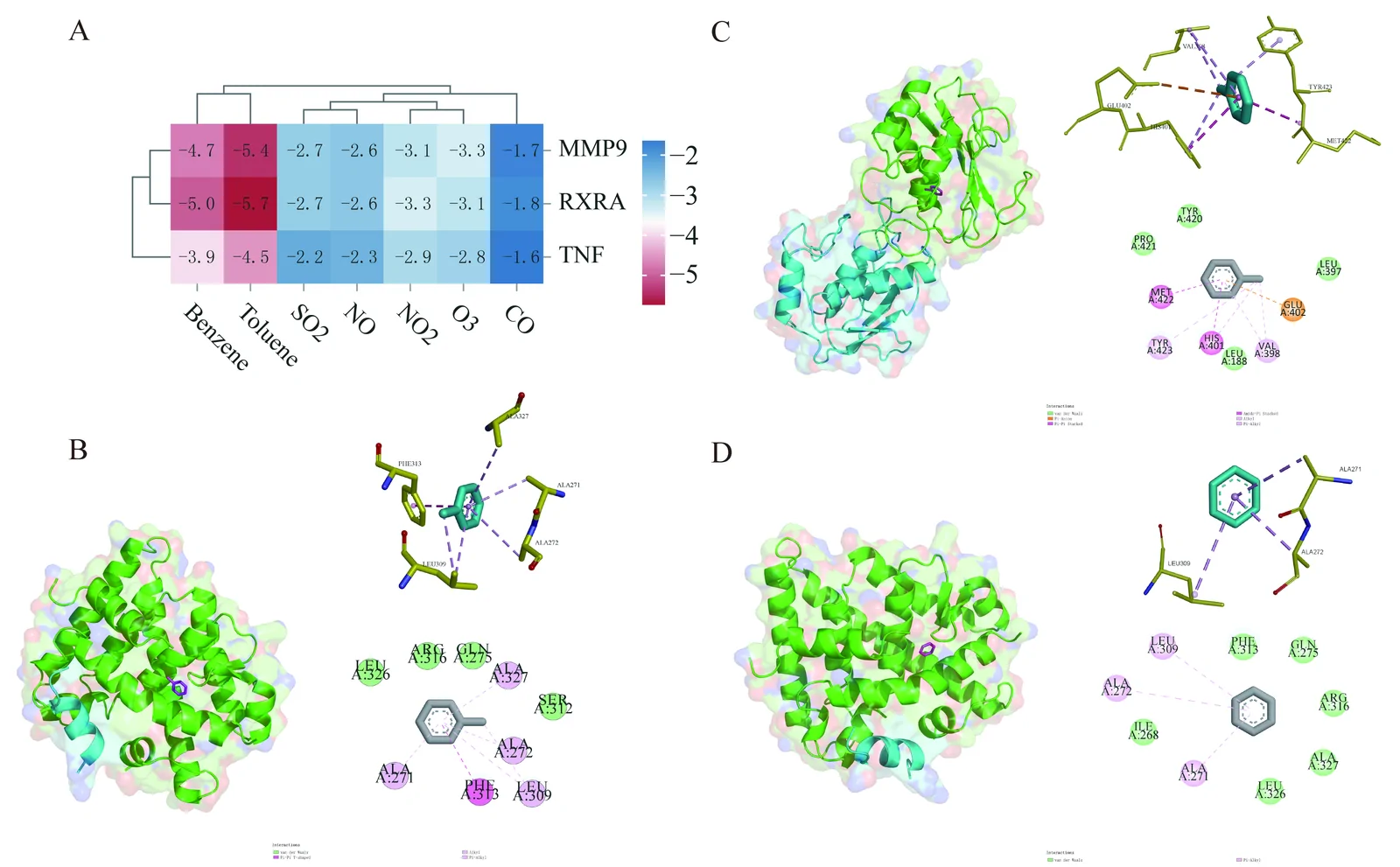
Molecular docking results. (**A**) Heatmap representing the docking binding free energies between core genes and seven gaseous pollutants; (**B**) Molecular docking of Toluene with *RXRA*; (**C**) Molecular docking of Toluene with *MMP9*; (**D**) Molecular docking of Benzene with *RXRA*.

We systematically assessed the binding interactions of seven gaseous pollutants with three core target proteins—*RXRA*, *MMP9*, and *TNF*—and their key downstream effectors using molecular docking. Our results revealed negative binding free energies for most protein-ligand pairs, indicating thermodynamically spontaneous binding. Notably, toluene exhibited strong binding affinity for both *RXRA* and *MMP9*, with binding free energies below −5 kcal/mol (Figure 4A), suggesting a potential for molecular interaction based on computational prediction. To further elucidate the binding mechanisms, we performed three-dimensional visualization of the top three conformational complexes with optimal binding energies (Figure 4B–D). Structural analysis demonstrated that the ligand molecules docked stably within the active sites or critical functional regions of the target proteins, forming multiple molecular interactions such as hydrophobic contacts and van der Waals forces.

### 2.6. Single-Cell Transcriptomics Reveals Cellular Distribution of Core Genes

**Figure 5:**
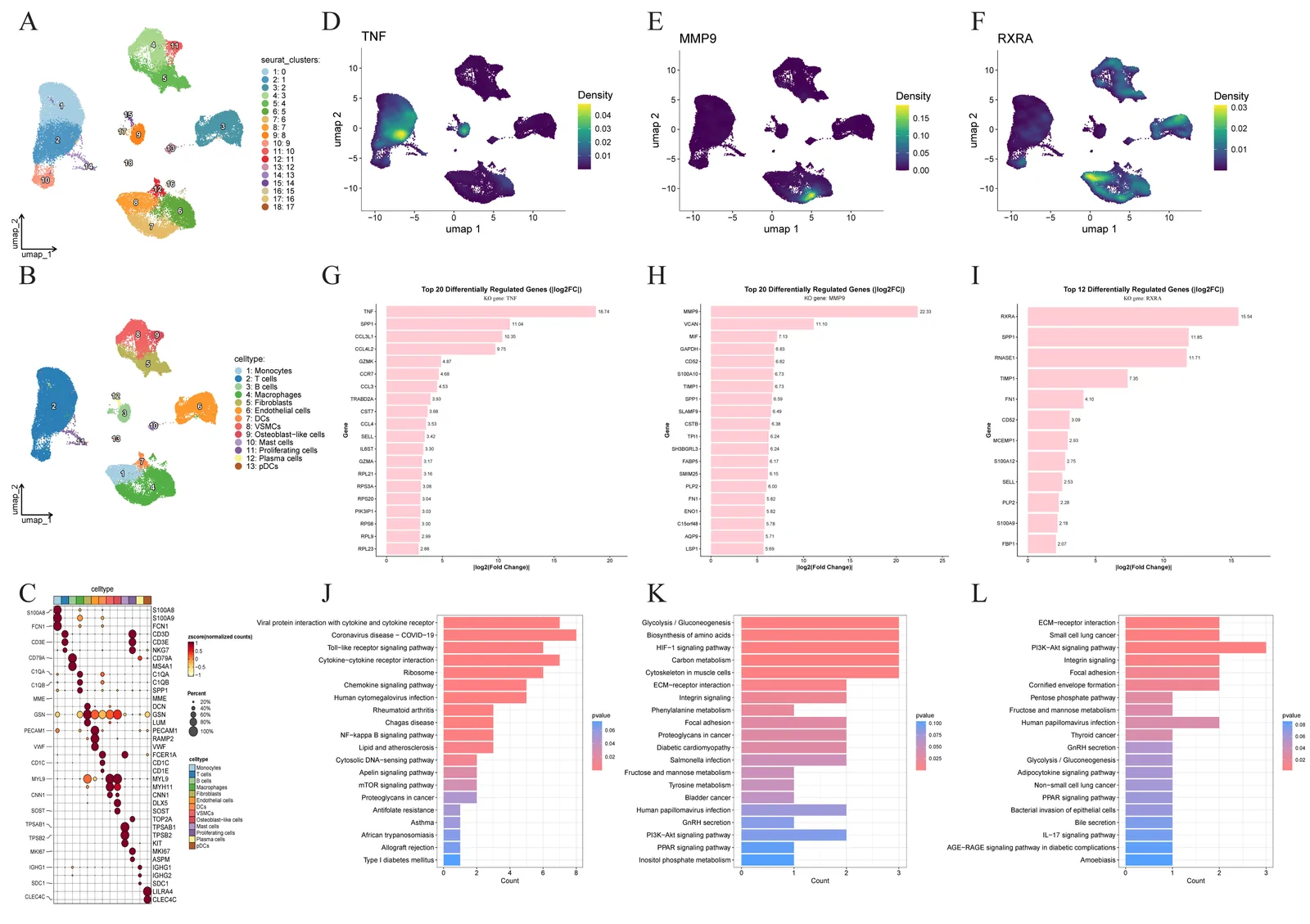
Single-cell RNA sequencing analysis. (**A**) UMAP plot showing 18 distinct cell clusters; (**B**) UMAP plot with annotation of 10 major cell types based on canonical lineage markers; (**C**) Bubble plot displaying expression levels of marker genes used for cell type identification; (**D**–**F**) Density plots showing the distribution of core gene expression across cell subtypes. *RXRA* was broadly expressed across multiple cell types, with enrichment in monocytes, macrophages, and endothelial cells. *MMP9* expression was predominantly restricted to macrophages, while *TNF* was mainly observed in T cells; (**G**–**I**) Bar plots showing significantly perturbed genes following virtual knockout of *RXRA*, *MMP9*, and *TNF*; (**J**–**L**) KEGG pathway enrichment analysis of the perturbed genes after knockout of *RXRA*, *MMP9*, and *TNF*.

To delineate the cellular distribution and expression patterns of atherosclerosis-associated genes at single-cell resolution, this study performed an integrated analysis of the public single-cell transcriptome dataset GSE159677, derived from human carotid artery plaques and adjacent non-plaque tissue. Through unsupervised clustering and identification using canonical lineage markers, cells from GSE159677 were classified into 18 subclusters and annotated as 10 major cell types (Figure 5A–C).

Further focusing on the expression patterns of three core genes—*RXRA*, *MMP9*, and *TNF*—revealed pronounced cell-type specificity. *RXRA* was broadly expressed across multiple cell types, with particular enrichment in monocytes, macrophages, and endothelial cells. In contrast, the expression of *MMP9* and *TNF* was highly restricted to immune cell populations: *MMP9* was predominantly observed in macrophages, while *TNF* was mainly found in T cells (Figure 5D–F).

To further investigate the potential functions of these cell-type-specifically expressed genes in atherosclerosis progression, this study conducted in silico knockout simulations for the three core genes in atherosclerosis-associated cell types, followed by KEGG pathway enrichment analysis, and visualized the significantly perturbed genes resulting from each gene knockout using bar plots (Figure 5G–I). The results demonstrated significant functional divergence among these genes in disease-related pathways, closely correlating with their cellular origins. Virtual knockout of *RXRA* was primarily enriched in metabolism-related pathways, such as the PPAR signaling pathway, adipocytokine signaling pathway, and glycolysis/gluconeogenesis, suggesting it may influence early lipid accumulation in atherosclerosis by regulating lipid metabolism and energy balance. Virtual knockout of *MMP9* was significantly enriched in pathways associated with extracellular matrix (ECM) remodeling and metabolic reprogramming, including ECM-receptor interaction, integrin signaling pathway, focal adhesion, as well as carbon metabolism and amino acid biosynthesis. Combined with its characteristic high expression in macrophages, this suggests that *MMP9* may participate in modulating plaque stability and the inflammatory microenvironment by regulating macrophage-mediated matrix degradation and metabolic adaptation. Virtual knockout of *TNF* primarily affected immune and inflammation-related pathways, such as the NF-κB signaling pathway and Toll-like receptor signaling pathway. Given its specific high expression in T cells, these results further support the role of T-cell-derived *TNF* as a key pro-inflammatory cytokine in atherosclerosis, driving local immune responses and inflammatory amplification (Figure 5J–L).

In summary, this study elucidates at the single-cell level the cellular origins and functional division of labor among *RXRA*, *MMP9*, and *TNF* in atherosclerosis: *RXRA* is broadly involved in the metabolic regulation of stromal cells, *MMP9* mediates ECM remodeling and metabolic reprogramming in macrophages, and *TNF* drives inflammatory signal amplification in T cells.

### 2.7. Spatial Mapping of Core Gene Expression in Atherosclerotic Plaques

To determine the spatial expression patterns of *RXRA*, *MMP9*, and *TNF* within atherosclerotic tissue architecture and validate our scRNA-seq-based cell localization predictions, we performed spatial transcriptomics on atherosclerotic tissue sections.

**Figure 6:**
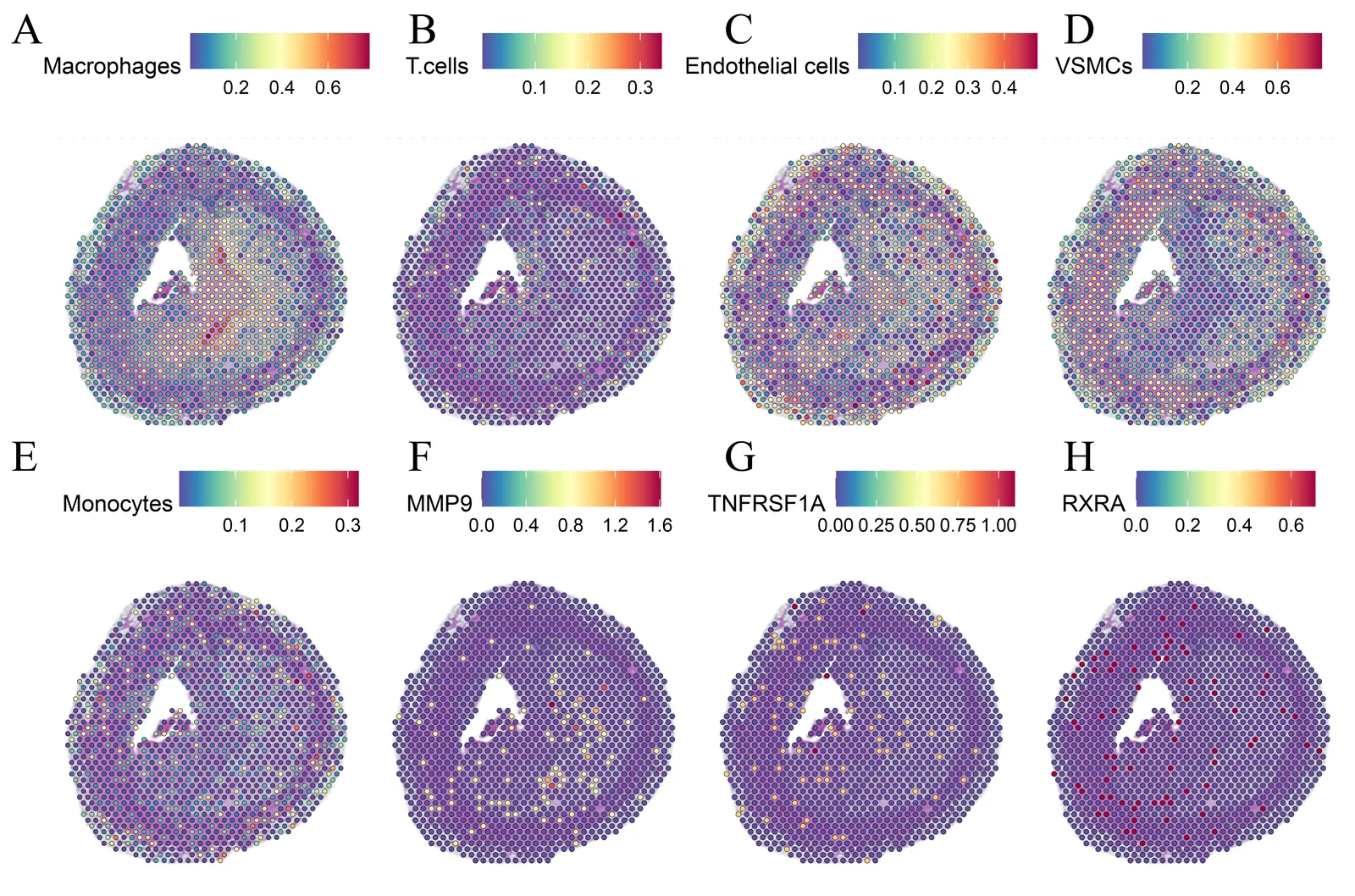
Spatial transcriptomic mapping of cell populations and core genes in atherosclerotic plaques. (**A**–**E**) Spatial distribution of major cell types—Macrophages, T cells, Endothelial cells, Vascular smooth muscle cells (VSMCs), and Monocytes—in atherosclerotic tissue, mapped using RCTD deconvolution; (**F**–**H**) Spatial expression patterns of core genes *RXRA*, *MMP9*, and *TNFRSF1A*. *MMP9* showed high expression in both plaque and necrotic core areas; *TNFRSF1A* was primarily expressed in plaque regions; *RXRA* exhibited prominent expression in the vascular intima and plaque regions.

Using spatial clustering of tissue regions, we identified and annotated major cell populations, including Endothelial cells, Monocytes, T cells, vascular smooth muscle cells (VSMCs), and macrophages, thereby delineating the spatial architecture of the lesioned tissue (Figure 6A–E).

Spatial mapping of the three core genes revealed distinct yet overlapping expression patterns: *MMP9* displayed high expression in both plaque and necrotic core areas (Figure 6F); *TNFRSF1A* was primarily expressed in plaque regions (Figure 6G); and *RXRA* showed prominent expression in the vascular intima and plaque regions (Figure 6H).

The high-expression regions of all three genes displayed substantial spatial overlap, collectively converging within the plaque area as the pathological epicenter. This spatial co-localization provides compelling transcriptional-level evidence that strongly validates the gene expression patterns derived from our single-cell analysis. Consequently, these results confirm the coordinated involvement of these core genes in atherosclerotic plaque pathogenesis.

### 2.8. Histomorphological and Serum Biomarker Analyses of Core Targets

To examine the spatial distribution and expression of the three core genes (*RXRA*, *TNF*, and *MMP9*) during atherosclerosis, we performed spatial transcriptomic profiling of clinical specimens from diabetic patients, who often present with comorbid atherosclerosis. Our analysis compared gene expression patterns between lesioned versus non-lesioned vascular regions within the same patients.

**Figure 7:**
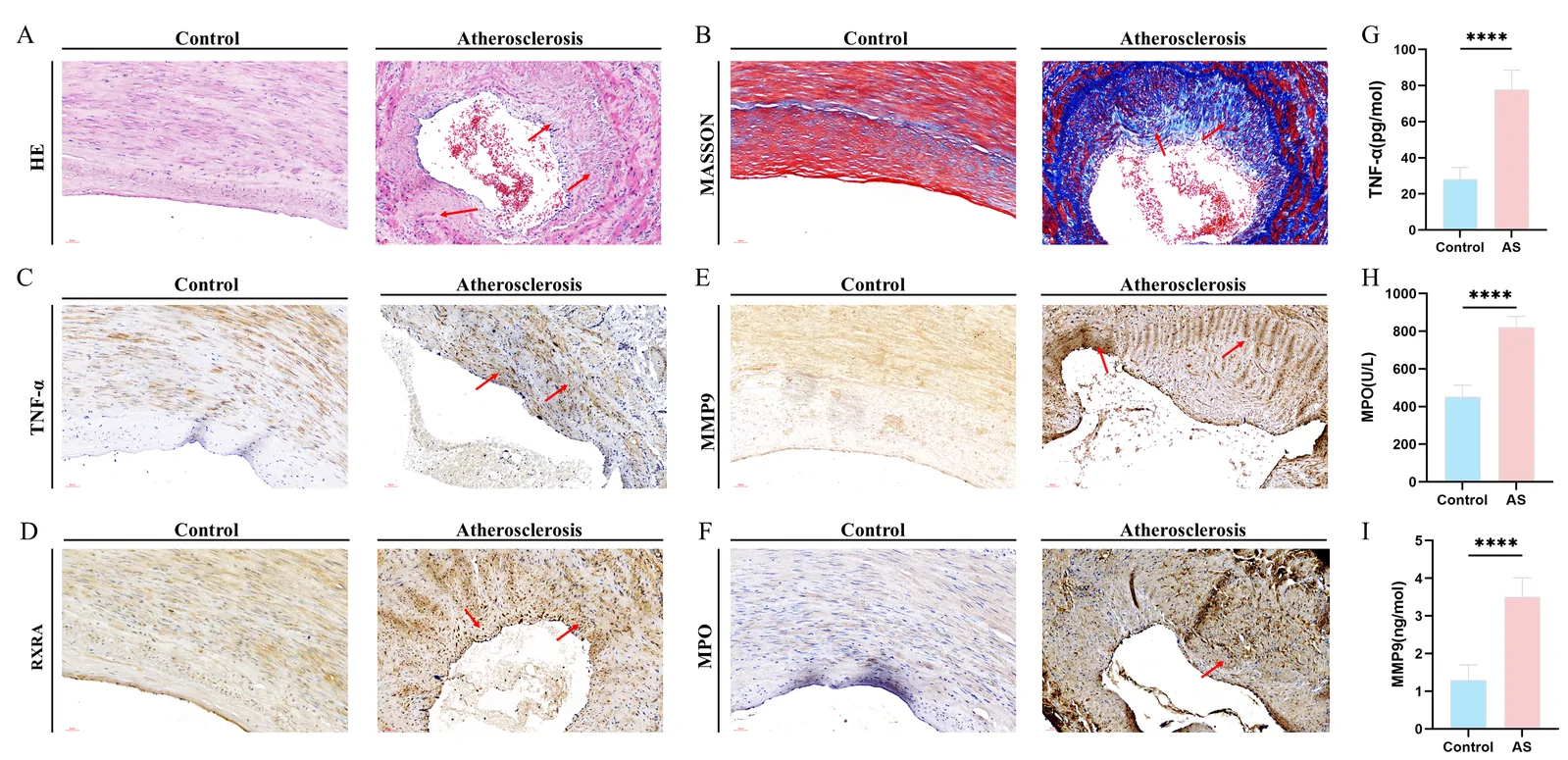
Clinical validation of core target expression in human atherosclerotic tissues and serum. (**A**,**B**) Representative H&E and Masson’s trichrome staining showing atherosclerotic vascular morphology; (**C**–**F**) Immunohistochemical staining of *TNF-α*, *RXRA*, *MMP9*, and *MPO* in non-lesioned versus atherosclerotic vascular tissues. All three proteins were markedly upregulated in atherosclerotic lesions, predominantly localized to plaque core regions, foam cells, and inflammatory infiltrates. Scale bars, 50 μm; (**G**–**I**) Serum levels of *TNF-α* (**G**), *MPO* activity (**H**), and *MMP9* (**I**) in healthy controls and atherosclerosis patients. *TNF-α* and *MMP9* were measured by ELISA; *MPO* activity was assessed using a specific activity assay kit. All three markers were significantly elevated in the patient group. Red arrows indicate the atherosclerotic plaque lesion areas. Data are mean ± SD. **** *p* < 0.0001.

Histological analysis of diseased vessels using H&E and Masson’s trichrome staining showed characteristic atherosclerotic alterations, including neointimal thickening, lipid deposition, and altered vessel morphology (Figure 7A,B).

Immunohistochemical analysis revealed markedly different expression patterns between non-lesioned and atherosclerotic tissues. In non-lesioned vascular segments, *RXRA*, *TNF*, and *MMP9* protein expression remained barely detectable, with preserved architecture of the intima, media, and adventitia. In contrast, atherosclerotic lesions exhibited marked upregulation of all three proteins. Under high-power magnification, strong positive staining for *RXRA*, *MPO*, *TNF*, and *MMP9* was predominantly localized to the core regions of atherosclerotic plaques and the diseased intima. Notably, positive signals were primarily observed in foam cells, inflammatory infiltrates, and some vascular smooth muscle cells within plaques, indicating a close association between these proteins and key pathological cellular components. IHC staining showed increased expression of these proteins in atherosclerotic lesions compared with non-lesioned areas, consistent with their involvement in atherosclerosis (Figure 7C–F).

To further assess the clinical relevance of the three core targets in atherosclerosis pathogenesis, we collected blood samples from patients with ultrasonographic evidence of carotid intima-media thickening (CIMT > 1.0), a surrogate marker of atherosclerosis, and quantified serum levels of *TNF* and *MMP9* using ELISA.

Our analysis demonstrated significantly elevated serum concentrations of *TNF* and *MMP9* in atherosclerosis patients compared with healthy controls. Similarly, serum *MPO* levels showed a marked increase in the patient group, consistent with the upregulation patterns observed for *TNF* and *MMP9* (Figure 7G–I).

These findings showed that circulating levels of *TNF* and *MMP9* are significantly elevated in atherosclerosis patients. Importantly, the concurrent increase in *MPO*—the upstream activator of *RXRA*—closely corresponds to our tissue-based observation of enhanced *RXRA* expression. The concurrent upregulation of *MPO* and *RXRA* observed in atherosclerotic tissues is consistent with the recognized role of systemic inflammatory mechanisms in this condition.

### 2.9. Toluene Upregulates RXRA, MMP9, and TNF-α Expression in M1 Macrophages

Based on immune infiltration and single-cell analyses, macrophages were identified as a key immune population in atherosclerotic lesions, and molecular docking indicated strong binding affinities between toluene and the candidate core proteins *RXRA*, *MMP9*, and *TNF-α*. Toluene was therefore selected as the representative pollutant for in vitro validation using THP-1-derived M1 macrophages.

**Figure 8:**
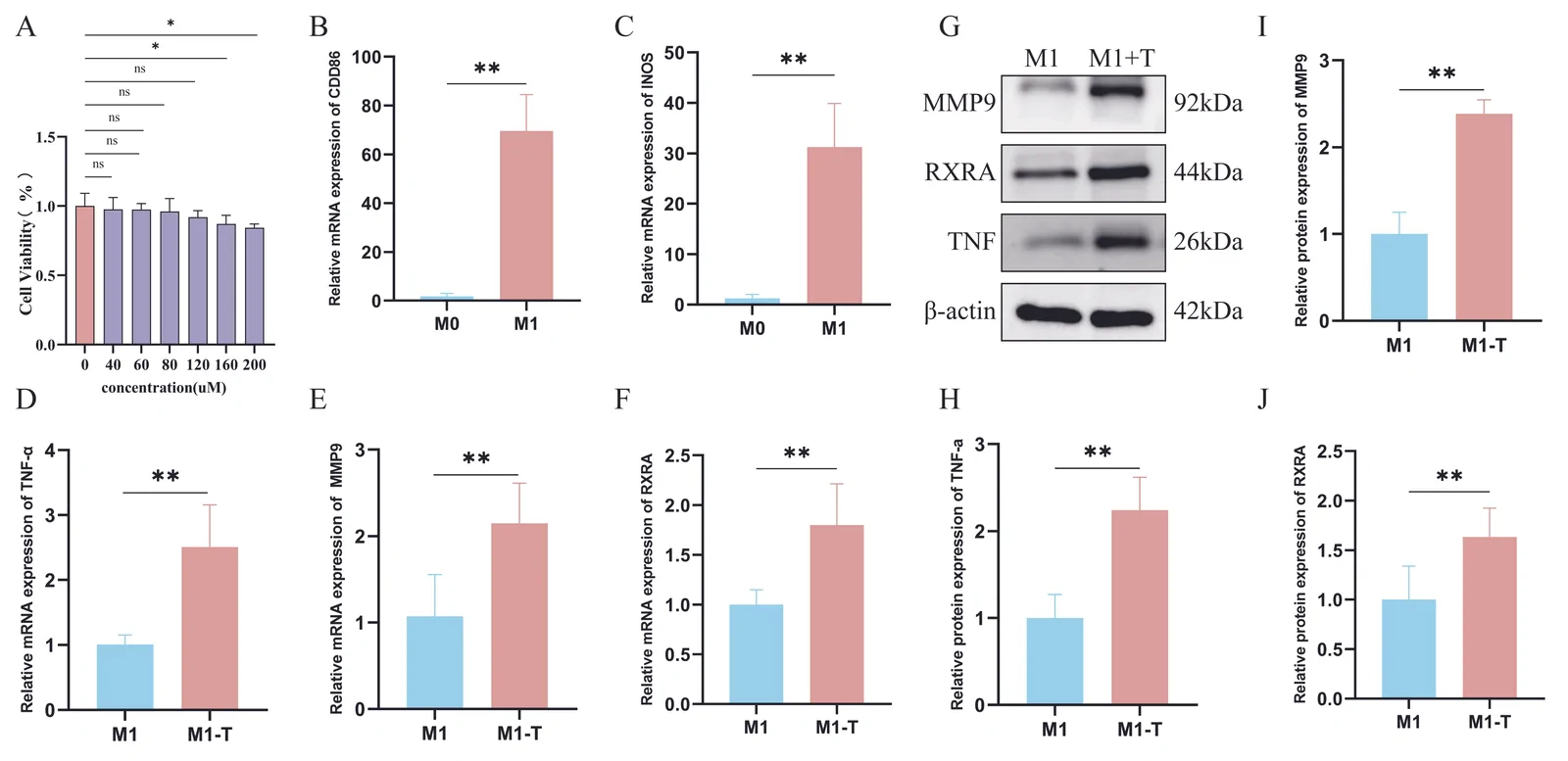
Toluene promotes *RXRA*, *MMP9*, and *TNF-α* expression in M1-polarized THP-1 macrophages. (**A**) CCK-8 assay showing the viability of M1-polarized THP-1 macrophages after exposure to different concentrations of toluene for 24 h. A concentration of 120 µmol/L was selected for subsequent experiments because it did not significantly reduce cell viability; (**B**,**C**) RT-qPCR validation of M1 macrophage polarization markers. Compared with M0 macrophages, IFN-γ/LPS-stimulated macrophages showed significantly increased mRNA expression of CD86 and iNOS, confirming successful M1 polarization; (**D**–**F**) RT-qPCR analysis showing that toluene exposure significantly upregulated the mRNA expression levels of TNF-α, MMP9, and RXRA in M1 macrophages; (**G**) Representative Western blot bands of RXRA, MMP9, TNF-α, and β-actin in M1 macrophages with or without toluene exposure; (**H**–**J**) Quantitative analysis of Western blot results showing increased protein expression levels of TNF-α, MMP9, and RXRA after toluene treatment. Data are presented as mean ± SD. * *p* < 0.05, ** *p* < 0.01, ns: not significant.

CCK-8 assay identified 120 µmol/L as the highest toluene concentration that did not significantly compromise M1 macrophage viability (Figure 8A), and this concentration was adopted for all subsequent exposures. Successful M1 polarization was confirmed by the significantly elevated mRNA levels of *CD86* and *iNOS* in IFN-γ/LPS-stimulated cells compared with M0 macrophages (*p* < 0.01; Figure 8B,C).

Exposure of M1 macrophages to 120 μmol/L toluene for 24 h significantly upregulated the mRNA expression of *TNF-α*, *MMP9*, and *RXRA* relative to the unexposed M1 group (all *p* < 0.01; Figure 8D–F). Consistently, Western blot analysis demonstrated markedly increased protein levels of MMP9, RXRA, and TNF-α (all *p* < 0.01; Figure 8G–J). These results showed that toluene enhances the expression of RXRA, MMP9, and TNF-α in M1 macrophages.

## 3. Discussion

By integrating network toxicology, multiple machine learning algorithms, multi-omics analyses, single-cell and spatial transcriptomics, and clinical tissue validation, this study delineates the coordinated roles of *RXRA*, *MMP9*, and *TNF* in gaseous air pollution-associated atherosclerosis. This integrative approach provides a systems-level understanding of the core pathological mechanisms through which environmental pollutants contribute to cardiovascular disease. In contrast to conventional research focusing on isolated pathways such as oxidative stress or inflammation, our findings establish a continuous evidence chain linking pollutant exposure to molecular targets, cellular responses, and tissue pathology. These insights highlight the convergent roles of metabolic and inflammatory processes in pollutant-promoted atherosclerosis, offering a comprehensive molecular basis for understanding environment-induced cardiovascular pathology.

From a pollutant-specific cardiovascular perspective, benzene, SO_2_, NO_2_, O_3_, NO, CO, and toluene may promote atherosclerosis through overlapping oxidative, inflammatory, and metabolic pathways. Benzene increases stroke risk via CYP450-mediated oxidative stress and indirect inflammatory priming [14]. SO_2_ elevates cardiovascular mortality and triggers endothelial dysfunction [15]. NO_2_, with the largest predicted target network, drives coronary risk not only through accelerated biological aging and macrophage-driven inflammatory senescence, but also via endothelial dysfunction, oxidative stress, and systemic inflammatory responses [16]. Long-term O_3_ exposure is associated with higher CVD incidence, likely mediated by pulmonary oxidative stress and secondary systemic inflammation [17]. Exogenous NO induces oxidative/nitrosative stress [18]. Although low endogenous CO may be anti-inflammatory [19], high-level environmental exposure causes hypoxic and arrhythmogenic effects via carboxyhemoglobin formation. Toluene, a component of BTEX (benzene, toluene, ethylbenzene, and xylene) primarily emitted from traffic and industrial sources, has been associated with cardiovascular hospitalization in epidemiological studies, particularly among occupationally exposed populations, along with dyslipidaemia and mild blood pressure elevation [20].

Multi-omics analyses consistently identified *RXRA*, *MMP9*, and *TNF* as central molecular hubs linking pollutant exposure to atherosclerosis progression. As a key nuclear receptor, *RXRA* serves a dual role in atherosclerosis pathogenesis: it regulates lipid metabolism through heterodimer formation and also modulates inflammatory signaling in vascular endothelial cells [21,22,23]. Our multi-omics data revealed widespread expression of *RXRA* in vascular smooth muscle cells, endothelial cells, and fibroblasts. GSVA further showed a strong positive correlation between *RXRA* and xenobiotic metabolism pathways, suggesting that *RXRA* functions as an early metabolic sensor in response to pollutant exposure. These findings, together with previous reports that PM2.5 exposure can induce dyslipidemia (reduced HDL-C and elevated LDL-C), suggest that *RXRA*-mediated metabolic alterations may participate in the early stages of atherosclerosis under pollutant exposure, complementing the known metabolic disturbances that precede inflammatory amplification [24,25]. Given that *RXRA* functions primarily as an intracellular nuclear receptor with undetectable circulating levels, we instead measured its key upstream activator—myeloperoxidase (*MPO*), a neutrophil-derived pro-inflammatory enzyme known to enhance nuclear receptor transcriptional activity, including *RXRA* [26].

Building upon this early metabolic activation, *MMP9* and *TNF* collaboratively drive the subsequent inflammatory escalation within atherosclerotic plaques. Single-cell and spatial transcriptomic analyses precisely localized their expression to macrophage-enriched regions, where both genes showed strong positive correlations with the infiltration of additional pro-inflammatory immune cells. Functionally, *TNF* facilitates inflammatory cell recruitment through activation of the NF-κB signaling pathway [27], whereas *MMP9* promotes extracellular matrix degradation and compromises fibrous cap integrity, consistent with its established role in plaque destabilization [28,29]. Moreover, spatial co-localization analyses demonstrated that *RXRA*, *MMP9*, and *TNF* converge to form integrated “inflammatory hotspots” within plaque cores, providing tissue-level evidence of their coordinated amplification in the disease microenvironment. The observed increase in activated mast cells is consistent with their known roles in promoting plaque inflammation and destabilization through the release of pro-inflammatory mediators and proteases, including *TNF* and *MMP9*.

Histological observations and serological measurements further suggest the pathophysiological relevance of *RXRA*, *MMP9*, and *TNF* in atherosclerosis. In vascular tissues from diabetic patients with atherosclerosis, protein expression of all three molecules was significantly upregulated and spatially correlated with pathological lesions. Serological analyses also confirmed elevated circulating levels of *MMP9*, *TNF*, and *MPO*—an upstream regulator of *RXRA*—in atherosclerosis patients. These findings align with previous studies indicating that *MPO* enhances *MMP9* activity and promotes inflammatory cascades. Collectively, these results integrate histological observations with systemic changes, suggesting cross-scale consistency in the expression and detectability of this molecular triad, aligning with the molecular signature identified in public cohorts.

To further strengthen the mechanistic connection between pollutants and these molecular targets, we performed molecular docking analysis and in vitro validation. Molecular docking showed that among the evaluated gaseous pollutants, toluene displayed relatively strong binding affinity toward *RXRA*, *MMP9*, and *TNF*, suggesting potential molecular-level selectivity rather than a nonspecific association with inflammatory pathways. More importantly, our in vitro experiments using THP-1-derived M1 macrophages demonstrated that toluene exposure significantly increased the mRNA and protein expression levels of *RXRA*, *MMP9*, and *TNF*. This finding provides direct cellular evidence that pollutant exposure itself can activate this core molecular axis under controlled experimental conditions, independent of inter-individual clinical heterogeneity or the complex in vivo microenvironment. Therefore, the pollutant–target relationship proposed in this study is supported not only by computational prediction, but also by functional cellular validation. Together, these results suggest that gaseous pollutants may specifically modulate the *RXRA*–*MMP9*–*TNF* axis, thereby amplifying the intrinsic inflammatory processes underlying atherosclerosis.

It should be noted that gaseous pollutants primarily enter the body via inhalation and do not necessarily come into direct contact with cardiovascular cells or atherosclerotic plaques. Their potential influence on the cardiovascular system is more likely mediated through indirect pathways, including the induction of local oxidative stress in the respiratory tract with release of pro-inflammatory cytokines such as *IL-6* and *TNF-α* into the circulation, leading to systemic low-grade inflammation [30,31]; the activation of pulmonary neural reflexes or the sympathetic nervous system, resulting in vasoconstriction and endothelial dysfunction [32]; and for certain lipophilic pollutants such as toluene and benzene, possible translocation across the alveolar–capillary barrier into the systemic circulation, although their actual blood concentrations are expected to be far lower than the in vitro exposure levels used in this study, and the physiological relevance of this pathway requires further validation [33,34].

This study has several limitations. First, causal pollutant–target relationships require in vivo validation beyond the current molecular docking and in vitro experiments. Second, the absence of individual-level exposure data restricts our findings to pollution-associated rather than exposure-quantified effects. Third, the histopathological and IHC analyses in this study are primarily exploratory and serve as supplementary evidence to support the in silico predictions, rather than providing independent clinical validation; the small sample size (*n* = 3 per group) limits statistical power and precludes any definitive conclusions regarding clinical utility or biomarker potential. Furthermore, the molecular changes observed in clinical samples represent common inflammatory and metabolic pathways in atherosclerosis, and cannot be specifically attributed to air pollution given the presence of potential confounders such as diabetes and other unmeasured risk factors. Fourth, the in vitro validation was limited to toluene; whether other gaseous pollutants exert similar effects on the *RXRA*–*MMP9*–*TNF* axis requires further investigation. Furthermore, the toluene concentration used in vitro was selected based on cellular tolerance rather than environmental relevance; this concentration exceeds typical ambient exposure levels and may activate pathways that are not operative at environmentally realistic concentrations. The in vitro findings should therefore be interpreted as proof-of-concept rather than direct representations of ambient exposure effects. Fifth, in this study, qPCR analysis used a single reference gene, GAPDH, for normalization. Although its stability under the experimental conditions was retrospectively verified, the possibility of pollutant-induced drift in reference gene expression cannot be completely ruled out. Future studies should employ ApoE^−^/^−^ or other in vivo models, incorporate prospective cohort designs with individual exposure monitoring, validate findings in larger clinical samples, assess the molecular effects of specific PM_2.5_ components, and use multiple reference genes to ensure accurate normalization.

## 4. Materials and Methods

### 4.1. Compilation of Air Pollutant-Associated Targets

We obtained molecular structures for seven gaseous pollutants from the PubChem database (https://pubchem.ncbi.nlm.nih.gov/, accessed on 23 July 2026) [35], which provided molecular formulas and SMILES identifiers. We selected these seven gaseous pollutants because their defined molecular structures enable reliable molecular docking and in vitro exposure modeling, which is challenging for heterogeneous particulate matter. Moreover, they serve as important precursors and co-pollutants of PM_2.5_, allowing the results to indirectly inform on the mechanisms of particulate matter toxicity. Using these structures, we systematically probed potential biological targets in humans through several online prediction platforms: TargetNet (structure-based prediction) (http://targetnet.scbdd.com/, accessed on 23 July 2026) [36], ChEMBL (experimentally validated interactions) (https://www.ebi.ac.uk/chembl/, accessed on 23 July 2026), STITCH (high-confidence interactions with combined score ≥ 0.7) (https://stitch.embl.de/, accessed on 23 July 2026) [37], and SuperPred (similarity-based prediction) (https://prediction.charite.de/, accessed on 23 July 2026) [38]. We integrated and standardized the target information collected from each database. After removing redundant entries, we obtained a final set of high-confidence putative targets. In this study, “targets” are defined as human genes or proteins that potentially interact with gaseous pollutants, as inferred from the above databases, prediction platforms, and supporting literature.

### 4.2. Identification of Differentially Expressed Genes in Atherosclerosis

We acquired the gene expression dataset GSE100927 from the GEO database. This dataset comprises transcriptomic profiles from 69 patients with atherosclerosis and 35 healthy controls. We retrieved raw expression matrices using the “GEOquery” R package and performed data preprocessing and differential expression analysis with the “limma” package. These steps included background correction, normalization, and gene symbol annotation. We defined statistically significant differential expression using thresholds of |log_2_FC| ≥ 0.5 and an adjusted *p*-value (Benjamini–Hochberg FDR) < 0.05. Finally, we visualized the results with a volcano plot generated by “ggplot2” and a heatmap created using the “pheatmap” package.

### 4.3. Construction of the Protein–Protein Interaction (PPI) Network

We imported the overlapping targets into the STRING database (https://stringdb.org, accessed on 23 July 2026) to construct a protein–protein interaction (PPI) network [39]. The search was limited to Homo sapiens, with a minimum interaction confidence score set at 0.4. To simplify visualization, we hid disconnected nodes. The resulting PPI network was subsequently imported into Cytoscape for further analysis [40]. Using the CytoNCA plugin, we assessed the topological importance of each node according to six centrality parameters: betweenness (BC), closeness (CC), degree (DC), eigenvector (EC), network (NC), and local average connectivity (LAC). We identified candidate core targets as nodes whose values exceeded the median for all six parameters.

### 4.4. Functional Enrichment Analysis: GO and KEGG Pathways

We performed functional enrichment analysis on the overlapping gene set using the clusterProfiler package in R. This analysis included Gene Ontology (GO) term annotation—covering biological process (BP), cellular component (CC), and molecular function (MF)—as well as pathway analysis based on the Kyoto Encyclopedia of Genes and Genomes (KEGG). We considered terms with a *p*-value < 0.05 statistically significant. For the GO results, we selected the top 10 most significant entries from each category (BP, CC, and MF) for visualization. Similarly, we retained the top 30 significantly enriched KEGG pathways. We visualized the enriched terms and pathways using bar plots and bubble plots generated with the ggplot2 package, sorted by *p*-value and gene count to highlight key biological themes.

### 4.5. Machine Learning-Based Identification of Core Genes

To identify key genetic targets in gaseous air pollution-induced atherosclerosis, we systematically applied nine distinct machine learning algorithms for feature selection. The methods comprised: Lasso (Least Absolute Shrinkage and Selection Operator), Support Vector Machine (SVM), Random Forest (RF), Generalized Linear Model (GLM), Decision Tree (DT), eXtreme Gradient Boosting (XGB), K-Nearest Neighbors (KNN), Neural Network (NNET), and Gradient Boosting Machine (GBM). We performed these analyses using the following R packages: Lasso via glmnet, SVM via e1071, RF via randomForest, GLM, KNN, and NNET via caret, DT via rpart, XGB via xgboost, and GBM via gbm. From each algorithm, we extracted the top ten feature genes ranked by importance. We then integrated the results from all nine methods to compile a candidate gene set. Finally, we visualized inter-algorithm overlaps using an UpSet plot generated with the UpSetR package to identify robust core genes consistently prioritized across multiple approaches.

### 4.6. Analysis of Immune Cell Infiltration

We employed CIBERSORT (https://cibersortx.stanford.edu/, accessed on 23 July 2026) [41], a deconvolution algorithm that estimates the relative proportions of immune cell types in heterogeneous tissue samples using gene expression data. Using this approach, we quantified the infiltration levels of various immune cells within the sample immune microenvironment. We compared immune cell proportions across different subtypes using the Wilcoxon rank-sum test, considering results with a *p*-value < 0.05 statistically significant. Subsequently, we performed Spearman correlation analysis to assess the relationships between the expression of identified hub genes and the extent of immune cell infiltration. Finally, we visualized all analytical results using the “ggplot2” package, generating correlation heatmaps and stratified scatter plots to illustrate significant associations.

### 4.7. Gene Set Variation Analysis (GSVA)

To delineate the metabolic regulatory pathways involving the identified core genes, we performed Gene Set Variation Analysis (GSVA). GSVA is a nonparametric, unsupervised method that evaluates the variation in enrichment activity of predefined gene sets across individual samples. Using the GSVA package in R, we quantified enrichment scores for 50 hallmark gene sets, with a statistical significance threshold set at *p* < 0.05. We further conducted Spearman’s rank correlation analysis to examine associations between core gene expression levels and the enrichment scores of significantly altered metabolic pathways. The resulting correlation matrix was visualized as a heatmap using the Pheatmap package.

### 4.8. Molecular Docking Validation

We retrieved the three-dimensional structures of the receptor proteins from the PDB database and validated them through UniProt. We prepared the receptor structures using Discovery Studio 2019 Client by removing water molecules and native ligands, and by defining the binding site grid box. Subsequently, we conducted further preprocessing in AutoDockTools 1.5.6, adding hydrogen atoms, assigning partial charges, and exporting the final structures in PDBQT format. For ligand preparation, we obtained molecular structures from PubChem and performed energy minimization using the MM2 force field to achieve the lowest-energy conformation. We then processed these ligands in AutoDockTools 1.5.6 by adding hydrogens, setting rotatable bonds, and converting them to PDBQT format. Using AutoDock Vina 1.1.2 [42], we performed molecular docking and selected the top three poses with the most favorable binding affinity values for subsequent interaction analysis and visualization in Discovery Studio 2019 Client.

### 4.9. Single-Cell RNA Sequencing Analysis

We downloaded single-cell RNA sequencing data (GSE159677) from the GEO database and processed them using the Seurat package (v5.3.0) in R. We applied quality control filtering to retain cells that contained between 200 and 5000 detected genes and exhibited less than 15% mitochondrial gene content. We then normalized and scaled the data using the ScaleData function before performing principal component analysis (PCA). Subsequently, we conducted dimensionality reduction and unsupervised clustering through an integrated workflow. This involved applying RunHarmony for batch correction, using FindNeighbors and FindClusters (at dim = 20) to identify 18 distinct cell populations, and executing RunUMAP for visualization. Finally, we visualized expression patterns of core genes of interest with the FeaturePlot function. We further employed the scTenifoldKnk R package to construct gene regulatory networks based on Single-cell expression data. By simulating the knockout of core genes, we assessed the regulatory perturbations they exerted on other genes within the network, thereby predicting the potential functions of these genes.

### 4.10. Spatial Transcriptomics

To resolve the spatial distribution of cell subpopulations and core genes, we analyzed the spatial transcriptomics (ST) dataset GSE243179. We processed both ST and Visium sample data using the Seurat R package to generate gene-by-spot count matrices. We normalized gene expression data with the SCTransform function to correct for technical variance. After performing dimensionality reduction through principal component analysis (PCA) and unsupervised clustering, we mapped the resulting cell clusters back onto the tissue image using the SpatialDimPlot function.

For cell-type deconvolution within each spot, we applied RCTD (Robust Cell Type Decomposition), leveraging an annotated single-cell RNA-seq dataset (GSE159677) from three human carotid artery plaque samples as reference. This approach enabled us to reconstruct a spatially resolved cell type map. Finally, we visualized the spatial expression patterns of core genes using the SpatialFeaturePlot function to provide comprehensive tissue-level localization of these key targets.

### 4.11. Morphological Analysis of Human Vascular Tissues

Vascular tissue specimens were obtained from patients undergoing elective vascular surgery in our hospital. The study was approved by the Institutional Ethics Review Board, and written informed consent was obtained from all participants. Samples were assigned to two groups based on comprehensive clinical and pathological evaluation.

Atherosclerosis group: Tissues were collected from patients with atherosclerotic lesions confirmed by imaging (ultrasonography, CTA, or DSA), intraoperative observation, and postoperative pathology. Sufficient fresh or paraffin-embedded vascular/plaque tissue was required for subsequent qPCR, Western blot, or immunohistochemistry.

Control group: Tissues were obtained from patients undergoing surgery for non-atherosclerotic conditions, with pathological confirmation showing no significant atherosclerotic changes. The anatomical sampling sites were matched to those of the atherosclerosis group to minimize site-specific bias.

Exclusion criteria for both groups included: insufficient sample quantity or inadequate preservation quality; incomplete clinical baseline or follow-up data; concurrent acute infection, active malignancy, severe autoimmune disease, severe liver or kidney dysfunction, or any condition likely to significantly perturb inflammatory and immune status; and ambiguous tissue origin or pathological diagnosis that precluded reliable grouping.

All samples were independently screened by two investigators according to the above uniform criteria to reduce heterogeneity. Following rigorous screening, six tissue specimens (three atherosclerotic and three control) that met all criteria were included for subsequent morphological and molecular analyses. All vascular tissue specimens were obtained from diabetic patients undergoing elective vascular surgery. Atherosclerotic samples were collected from lesioned vascular segments, while control samples were obtained from adjacent or anatomically matched non-lesioned segments within the same patient group, as confirmed by postoperative pathology. This within-group comparison partially controlled for the systemic effects of diabetes, although residual confounding cannot be excluded.

All patients had long-term residency in Tianjin, a city located in the Beijing–Tianjin–Hebei region, where previous epidemiological studies have documented long-term exposure to ambient gaseous pollutants including PM_2.5_, O_3_, and NO_2_, which are associated with cardiovascular disease risk. Therefore, from a regional perspective, the included patients can be considered as deriving from a population with potential gaseous air pollution exposure [43,44].

We immediately fixed dorsal pedal artery tissue specimens in 4% paraformaldehyde following resection. Subsequently, we dehydrated the samples through a graded ethanol series, cleared them in xylene, and embedded them in paraffin blocks. Using a microtome, we sectioned the samples at 5 μm thickness. For histological evaluation, we deparaffinized and rehydrated the sections before staining them with hematoxylin and eosin (H&E) for general morphological assessment and with Masson’s trichrome for collagen quantification. Finally, we examined all stained sections under a Leica E100 light microscope to evaluate histopathological injury and collagen fiber content.

To evaluate the expression of core targets in clinical vascular tissues, we first washed deparaffinized and rehydrated sections three times with phosphate-buffered saline (PBS). We performed antigen retrieval by high-pressure heating in sodium citrate buffer (P0085, Beyotime) for 30 min, followed by cooling at room temperature for 1 h. Subsequently, we blocked endogenous peroxidase activity by incubating sections with 3% H_2_O_2_ for 10 min. We then incubated the sections with appropriately diluted primary antibodies at 4 °C overnight. The primary antibodies used were: anti-*MMP9* (rabbit polyclonal, 10375-2-AP, Proteintech), anti-*TNF-α* (rabbit polyclonal, 17590-1-AP, Proteintech), anti-*RXRA* (rabbit recombinant monoclonal, 83796-5-RR, Proteintech), and anti-*MPO* (rabbit polyclonal, 22225-1-AP, Proteintech). All primary antibodies were used at dilutions recommended by the manufacturer. After washing with PBS, we applied HRP-conjugated goat anti-rabbit IgG secondary antibody (DS-0004, ZSGB-BIO) and incubated at 37 °C for 20 min. This was followed by color development using DAB substrate (ZLI-9017, ZSGB-BIO). Finally, we counterstained the sections with hematoxylin, dehydrated, cleared, and mounted them. We digitally scanned all stained sections using a fully automated slide scanning system (WISLEAP) for subsequent analysis.

### 4.12. Measurement of Circulating Biomarker Levels in Human Blood Samples

We measured serum levels of tumor necrosis factor-α (*TNF-α*) and matrix metalloproteinase-9 (*MMP-9*) using ELISA kits (*TNF-α*: Boster, EK0525; *MMP-9*: Elabscience, E-EL-H6075). We assessed myeloperoxidase (*MPO*) activity with a specific activity assay kit (Elabscience, E-BC-K074-M) to evaluate neutrophil recruitment. All procedures followed the manufacturer’s instructions. We measured absorbance values using a microplate reader and calculated analyte concentrations against standard curves, with all samples analyzed in duplicate.

### 4.13. Cell Culture

The human monocytic leukemia cell line THP-1 (CL-0233, Pricella Life Science & Technology Co., Ltd., Wuhan, China) was cultured in RPMI-1640 (11875093, Gibco, Waltham, MA, USA) complete medium supplemented with 10% fetal bovine serum (FBS), 0.05 mM β-mercaptoethanol, and 1% penicillin/streptomycin, and maintained at 37 °C in a humidified incubator with 5% CO_2_. To generate M0 macrophage-like cells, THP-1 monocytes were seeded at a density of 5 × 10^5^ cells/mL and treated with 100 ng/mL phorbol 12-myristate 13-acetate (HY-18739, MCE, Shanghai, China) for 24 h. Subsequently, the medium was removed, and the adherent cells were washed once with pre-warmed phosphate-buffered saline (PBS) and incubated in fresh complete medium without PMA for an additional 24 h to allow full differentiation into resting M0 macrophages. For M1 polarization, the resulting M0 macrophages were stimulated with 20 ng/mL interferon-γ (UA040053, UA BIOSCIENCE, Nanjing, China) and 100 ng/mL lipopolysaccharide (HY-D1056, MCE) for 24 h. The M1-polarized macrophages were then used for subsequent toluene exposure experiments.

Toluene (8028LC0500, CONCORD) was first dissolved in dimethyl sulfoxide (DMSO) to prepare a stock solution, which was then diluted in RPMI-1640 complete medium to the desired working concentration. The final DMSO concentration in all treatment groups, including the vehicle control, was maintained at ≤0.1% (*v*/*v*) to avoid solvent-induced cytotoxicity. During toluene exposure, culture plates were sealed with parafilm to minimize evaporation of the volatile compound. All exposure experiments were performed in a dedicated incubator with temperature maintained at 37 °C.

### 4.14. Cell Viability Assay

Cell Counting Kit-8 (CCK-8) assay was performed to measure the viability of M1-polarized THP-1 macrophages upon toluene exposure. Briefly, M1 macrophages, obtained by sequential PMA differentiation and IFN-γ/LPS polarization in 96-well plates as described above, were treated with toluene at series concentrations (0, 40, 80, 120, 160, 200 μmol/mL) for 24 h. Subsequently, 10 μL of CCK-8 solution (HY-K0301, MCE) was added to each well and incubated at 37 °C for 30 min. Absorbance was measured at 450 nm using a microplate reader. The cell viability percentage was calculated as (OD_treatment − OD_control)/(OD_normal − OD_control) × 100%, where OD_treatment represents wells with toluene-treated M1 macrophages, OD_normal represents untreated M1 macrophages (vehicle control), and OD_control represents wells containing only culture medium and CCK-8 solution without cells.

### 4.15. Real-Time qPCR

Total RNA was extracted from toluene-treated M1 macrophages using a total RNA extraction kit (R1200, Solarbio, Beijing, China) according to the manufacturer’s instructions. RNA concentration and purity were determined with a micro-volume spectrophotometer; only samples with an A260/A280 ratio between 1.8 and 2.1 were used for subsequent reverse transcription. Equal amounts of total RNA were reverse-transcribed into cDNA using a FastKing RT Kit (KR118-02, TIANGEN, Beijing, China). Real-time PCR was performed with a Talent qPCR PreMix (SYBR Green) (FP205-01, TIANGEN) on a real-time PCR system. Each sample was run in triplicate. The primer sequences were as follows: *MMP9* (human): F: CTGGACAGCCAGACACTAAAG, R: CTCGCGGCAAGTCTTCAGAG, *TNF-a* (human): F: CAGAGGGCCTGTACCTCATC, R: GGAAGACCCCTCCCAGATAG, *RXRA* (human): F: TAGTCGCAGACATGGACACC, R: GTTGGAGAGTTGAGGGACGA, *iNOS* (human): F: CAGGGTGGAAGCGGTAACAAAGG, R: CCTGCTTGGTGGCGAAGATGAG, *CD86* (human): F: TGGAGAGGGAAGAGAGTGAACAGAC, R: AAACACGCTGGGCTTCATCAGATC, GAPDH (human): F: AGATCCCTCCAAAATCAAGTGG, R: GGCAGAGATGATGACCCTTTT, Finally, the mRNA expression levels of the relevant genes were calculated using the 2^−ΔΔCt^ method. GAPDH was used as the endogenous reference gene. The stability of GAPDH was retrospectively verified by analyzing the raw Ct values across all experimental groups; the intra-group coefficient of variation (CV) was less than 5% in each group, indicating acceptable stability. However, it should be noted that the use of a single reference gene under xenobiotic pollutant exposure conditions has inherent limitations.

### 4.16. Western Blotting

Western blotting was performed to detect the expression levels of RXRA, MMP9, and TNF proteins in M1-polarized THP-1 macrophages after toluene exposure. Total protein was extracted using RIPA (P0013B, Beyotime, Shanghai, China) lysis buffer containing 1% PMSF on ice and centrifuged to collect the supernatant. Protein concentration was determined by BCA assay (P0009, Beyotime). Equal amounts of protein were separated by SDS-PAGE and transferred to a 0.45 μm PVDF membrane. The membrane was blocked with 5% non-fat milk at room temperature for 2 h, then incubated with primary antibodies against RXRA (1:1000), MMP9 (1:1000), TNF (1:1000), and β-actin (1:10,000) overnight at 4 °C. After washing three times with TBST, the membrane was incubated with HRP-conjugated secondary antibody (7074S, CST, Shanghai, China) at room temperature for 80 min, followed by three additional TBST washes. Protein bands were visualized using an ultra-sensitive ECL chemiluminescence kit (ZD310, ZOMANBIO, Beijing, China). Grayscale values were quantified with ImageJ software (version 1.54), and the relative expression levels of target proteins were calculated after normalization to β-actin.

### 4.17. Data Analysis and Statistics

All statistical analyses were performed using R (version 4.4.3). Continuous variables are expressed as mean ± standard deviation (SD). Transcriptomic differential expression was assessed with limma (Benjamini–Hochberg adjusted *p* < 0.05). GO and KEGG enrichment analyses used hypergeometric tests (*p* < 0.05). Immune cell proportion comparisons were made using the Wilcoxon rank-sum test, and correlations were evaluated with Spearman’s rank test. For ELISA, qPCR, and Western blot data, two-group comparisons employed independent *t*-test or Mann–Whitney U-test as appropriate, and multi-group comparisons used one-way ANOVA followed by Tukey’s HSD post hoc test. A *p*-value below 0.05 was considered statistically significant. Statistical graphs were generated using GraphPad Prism (version 9.0), and schematic illustrations were created with BioRender.

## Data Availability

All data generated and analyzed in this study are included in this article. Data will be made available on request.

## Supplementary Materials

The following supporting information can be downloaded at: https://www.mdpi.com/article/10.3390/ijms27167381/s1.
